# Effectiveness of Integrated Maternal Nutrition Intervention Package on Birth Weight in Rwanda

**DOI:** 10.3389/fnut.2022.874714

**Published:** 2022-07-22

**Authors:** Michael Habtu, Alemayehu Gebremariam Agena, Maryse Umugwaneza, Monica Mochama, Cyprien Munyanshongore

**Affiliations:** ^1^College of Medicine and Health Sciences, School of Public Health, University of Rwanda, Kigali, Rwanda; ^2^Department of Public Health, School of Health Sciences, Mount Kenya University, Kigali, Rwanda; ^3^Catholic Relief Services, Chief of Party, Kigali, Rwanda

**Keywords:** birth weight, effectiveness, integrated nutrition intervention, low birth weight, Rwanda

## Abstract

Inadequate maternal nutrition before and during pregnancy is a principal risk factor for poor fetal development, resulting in low birth weight (LBW) and subsequently, poor child growth. Most studies focus on the impact of nutritional interventions after birth while only a few interventions consider integrated nutrition service packages. Therefore, there is limited evidence on whether integrated maternal nutrition interventions have a positive effect on birthweight. Thus, a post-program quasi-experimental study was carried out to determine the effectiveness of the integrated maternal nutrition intervention package on low birth weight in Rwanda. A total of 551 mother–baby pairs from the intervention and 545 controls were included in the analysis. Data regarding socio-demographic, maternal anthropometric parameters, and dietary diversity were collected using a structured questionnaire. Birth weight was assessed right after delivery, within 24 h. Logistic regression, linear regression, and path analysis were fitted to determine the effectiveness of the intervention on birth weight. The study found that the intervention reduced LBW by 66.99% (*p* < 0.001) and increased average birth weight by 219 g (*p* < 0.001). Logistic regression identified reduced risk of LBW among the intervention group (AOR = 0.23; 95%CI = 0.12–0.43; *p* < 0.001). It was also observed that the direct effect of the intervention on birth weight was 0.17 (β = 0.17; *p* < 0.001) and the main indirect mediator was maternal MUAC (β = 0.05; *p* < 0.001). Moreover, maternal passive smoking exposure and MUAC < 23 cm were found as risk factors for LBW. This study has demonstrated that an integrated maternal nutritional intervention package can significantly reduce LBW in low-income settings and should, therefore, be considered to improve birth weight.

## Introduction

Birth weight is a significant predictor of the present and future health status of a newborn, and low birth weight (LBW <2,500 grams) is a major public health concern (1, 2). Globally, 15–20% of newborns have low birth weight (3), which represents approximately 30 million newborns (23.4% of all births) every year (4, 5). Almost half of all LBWs are from South Central Asia (27%) and sub-Saharan Africa (15%) (4, 6, 7). In some African countries, the prevalence of LBW has been reported to range between 10 and 15.7% (8). In Rwanda, according to the Demographic and Health Survey, it is estimated at 7% (9).

Low birth weight remains the single most important predictor of infant morbidity and mortality globally (10, 11). In African countries, 30% of all deaths of infants are attributed to LBW (12). Babies with LBW are about 40 times more likely to die within the first 30 days of life compared to their counterparts with normal birth weight (13). Furthermore, it is a significant factor associated with higher probabilities of infection; malnutrition; susceptibility to childhood illnesses; long-term physical and mental disorders; and problems related to behavior, learning, and psychosocial development during childhood (1, 13–15).

Maternal malnutrition during pregnancy is considered to be the main determinant of LBW (16, 17). However, the damage caused by maternal undernutrition can be prevented by improving the nutritional status of pregnant women. Appropriate nutrition intervention strategies during pregnancy are key to improving maternal nutrition and the birth weight of their newborns (18). The *Lancet* Maternal and Child Nutrition series in 2013 proposed nutrition-specific and nutrition-sensitive interventions to improve fetal nutrition and for optimal growth and child development (19, 20).

In line with the *Lancet* series and Rwanda National Food and Nutrition Policy for 2013–2018 (21), a program by the name *Gikuriro* meaning “good growth” was implemented between 2016 and 2020. The intervention was funded by USAID and implemented by Catholic Relief Services in partnership with the Government of Rwanda, the Netherlands Development Organization and other Rwandan Civil-Society Organizations. The program targeted five districts, namely, Kayonza and Ngoma from Eastern Province, Nyabihu from Western Province, and Kicukiro and Nyarugenge from Kigali City, and they were selected because of malnutrition and the absence of development partners, as of late 2014. The program implemented key nutrition-specific and nutrition-sensitive interventions to prevent maternal and early childhood malnutrition. Among the nutrition-specific interventions were nutrition education and counseling, and the key nutrition-sensitive interventions included the promotion of increased agricultural productivity; economic strengthening through social safety nets; and improved access to Water, Hygiene, and Sanitation (WASH) services. Adequate nutrition has been well recognized as important for maternal and fetal health. However, the effects of such integrated interventions during pregnancy on the child’s nutritional outcomes, including birth weight, are yet to be ascertained in the context.

The majority of the studies previously conducted to determine the effectiveness of individual interventions in the first 1,000 days window period revealed a modest impact on linear growth (22). For example, a single intervention during pregnancy showed a 50-g difference in birth weight and a 15% reduction of LBW, reported as the biggest effect (23, 24). Another study also showed that balanced energy and protein supplementation among pregnant women can increase birth weight by 41 g (25). A recent systematic review that included 16 high-quality randomized controlled trials demonstrated that improved maternal nutritional status leads to a significant reduction in LBW (3). This was particularly more effective when the intervention is a combination of nutrition education and multi-micronutrient supplements (3). However, scientific evidence on the effect of an integrated nutritional intervention package during pregnancy for improving birth weight is scarce. Therefore, the purpose of this study was to examine the effectiveness of an integrated maternal nutrition intervention package on low birth weight in Rwanda.

## Materials and Methods

### Study Design and Settings

The study adopted a quasi-experimental design and was conducted from November 2020 to June 2021. It was a post-intervention evaluation. The intervention group was drawn from two districts namely Kayonza District (rural area) and Kicukiro District (urban area), where the integrated nutrition intervention package (nutrition-specific and nutrition-sensitive) was implemented. The selection criteria for the two districts were based on the high proportion of food insecurity according to the Comprehensive Food Survey and Vulnerability Analysis (26) and locality (rural vs. urban). Similarly, in selecting the control districts three criteria were applied: high food insecurity, no existing nutrition-sensitive and nutrition-specific intervention package, and settlement pattern (rural vs. urban). After considering all the criteria, Gisagara District (rural area) and Gasabo District (urban area) were selected as the comparison control group. In a district, one public district hospital and all health centers were included in the study.

### Study Interventions

#### Description of the Nutrition-Specific Intervention

The component of the intervention package, which is nutrition-specific, was nutrition education and counseling. The women received nutrition education and counseling by Community Health Workers (CHWs) and nutritionists. First, the nutritionists and CHWs in charge received training on the counseling guide module. Then, the CHWs in charge in turn trained the CHWs at the village level. The trained nutritionist counseled the pregnant women about nutrition during regular antenatal care visits and sessions lasted about 30 to 45 min each. Moreover, the nutritionists also trained the women through cooking demonstrations about a balanced diet through Village Nutrition School. In addition to these, the CHWs gave further nutrition education and counseling at the household level. The CHWs also received in-service training on a monthly basis. The main contents of the educational and counseling guide are indicated in Supplementary Material. The control group, on the other hand, only received routine nutritional education and counseling as per the Rwanda national ante-natal care guidelines adopted from WHO (27).

#### Description of the Nutrition-Sensitive Intervention

In this intervention, three components were implemented, including promotion of increased agricultural productivity, promotion of financial literacy/economic strengthening, and improved access to WASH services. (1) Increased agricultural productivity: Beneficiaries were taught to practice nutrition-sensitive agriculture and to increase agriculture production using of Bio-Intensive Agriculture Techniques (BIATs). They were grouped into Farmer Field Learning School (FFLS) and advised on how to improve production mainly to attain food security at the household level. (2) Promotion of financial literacy/economic strengthening: The economic status of the women was improved by grouping them into Saving and Internal Lending Communities (SILC) as a way of responding to financial problems that prevent them from attaining better nutritional outcomes. The main methods of SILC were as follows: training those in charge of economic strengthening and project coordinators and district cooperative staff; then the trained staff train the sector cooperative officers, where, they in turn train field agents from the community. Then the field agents sensitize the people about SILC and form the groups. The goal was to help these women better manage their existing resources by teaching them basic financial management skills. This enabled the poor to build up useful lump sums without incurring excessive debt or interest charges. (3) Water, Sanitation, and Hygiene (WASH) interventions: Improved WASH services were implemented using Community Based Environmental Health Promotion Program (CBEHPP) approach through Community Health Clubs (CHC) at the village level. CBEHPP is a hygiene behavior change approach to reach communities and empower them to identify their personal and domestic hygiene needs. CHC and a demonstration site in every village were formed and initiated. The CHCs were responsible for ensuring that the levels of hygiene were monitored, together with the CHW facilitator, who visited each household to observe the household sanitation and environmental conditions. A detailed description of these interventions is presented in Supplementary Material. However, the control group did not receive any of these nutrition-sensitive interventions.

### Study Population, Sampling, and Sample Size

The target population was mother–newborn pairs. All pregnant women who came for delivery to all public health facilities in the selected districts were recruited consecutively using the following inclusion criteria: (1) being a permanent resident in the study area and aged between 15 and 49 years, (2) having been enrolled in the selected nutrition intervention package at least 1 year before pregnancy and continued until delivery for intervention group but not for the control group, (3) belonging to wealth category (1 and 2, 4) those without any known medical, surgical, or obstetric problems/conditions, and (5) with live singleton babies and normal spontaneous delivery.

**FIGURE 1:**
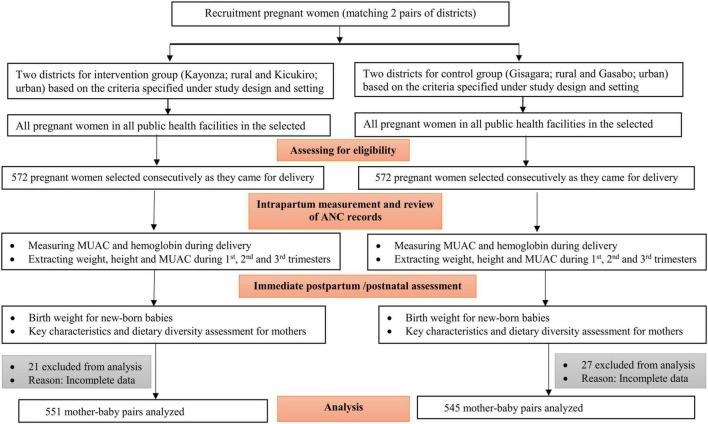
Recruitment flow chart.

The sample size was justified based on a 3.5% effect size and LBW proportion difference between the intervention and control group. This was estimated to detect a reduction of low birth weight from an expected 7% (9) in the control group (general population) to 3.5% in the intervention group. A power of 80%, a confidence level of 95%, and a design effect of 1.25 were considered to achieve the desired sample size. Thus, a total sample size of 1,144 (572 mother–newborn pairs for each study group) was estimated. However, 21 from the intervention and 27 from the control group were excluded from the analysis due to incomplete data. The recruitment flow chart is shown in Figure 1.

### Data Collection and Measurements

Trained midwives/nurses collected the data using a structured quantitative questionnaire. It was composed of basic demographic, lifestyle and obstetric factors, anthropometric and biological measurements, and maternal dietary diversity. To make data collection practices consistent, a standardized operating procedure was developed for all measures. The mothers were interviewed face-to-face in the immediate postpartum period (within 24 h of delivery) using a questionnaire that was translated into the local language (*Kinyarwanda*). The data collectors were trained on the objectives of the study, participants’ recruitment, and anthropometric measurements.

Anthropometric measurements were taken for both the mothers and their newborns. The nutritional status of the mothers was assessed using mid-upper arm circumference (MUAC), body mass index (BMI), and weight gain. MUAC has been identified to have a strong relationship with LBW and it is not affected by any changes like edema common during pregnancy (28). It was measured using flexible non-elastic tape upon recruitment. On the basis of several studies in Africa and due to the need for international comparison, maternal undernutrition was defined as MUAC < 23 cm (28). In addition, antenatal care records were reviewed to retrieve MUAC, weight, and height which are measured during the ANC visits according to Rwandan Ministry of Health guidelines. Weight and height were used to assess body mass index (BMI) and weight gain.

Hemoglobin was measured upon recruitment before delivery using a portable HEMOCUE B-Hb photometer according to Rwandan Ministry of Health guidelines. For the newborn babies, weight was measured within 24 h of delivery. The primary outcome of the study was birth weight. It was measured to the nearest 100 g on digital scales at the health facilities. The scales were regularly calibrated as per the manufacturer’s recommendation. Low birth weight was defined as birth weight less than 2,500 g.

A food frequency questionnaire was also used to obtain dietary information. This tool included 9 food groups validated by the Food and Agriculture Organization (29). These food groups were cereals and tubers; pulses and legumes; vegetables; fruits; meat, fish and eggs; milk and milk products; oils and fats, and sweets and spices/beverages. Pregnant women presenting to the health facilities for delivery were asked about their frequency of food consumption based on these groups. They were asked to recall what they had eaten in the 24 h preceding the onset of labor. A dietary diversity score was then calculated according to the frequency of food groups consumed by women within the 24 h. A score “1” was assigned to each consumed food group and a score “0” was assigned if not consumed. The scores were aggregated to calculate the total maternal dietary diversity score (DDS) and those who scored below 5 were grouped as inadequate DDS whereas those who scored 5 and above were categorized as having adequate DDS.

### Data Analysis

The analysis was performed using Statistical Package for the Social Sciences (SPSS) version 25.0 IBM New York. Means and percentages were used to summarize continuous and categorical data, respectively. Comparison of the explanatory variables between the intervention and control groups were conducted using an independent sample *t*-test (to compare means) or Chi-square test (to compare proportions). All statistical significance was set at a *p*-value less than 0.05.

The dependent variable was categorized into LBW (<2,500 g) and normal birth weight (≥2,500 g). To assess the effectiveness of the intervention, logistic regression was conducted by considering all variables with *p* < 0.2 in the bivariate analysis. The strength of association between LBW and the intervention was presented using an adjusted odds ratio with a corresponding 95% confidence interval. The model fitness was checked using the Hosmer and Lemeshow Test (*p*-value = 0.214), which indicated the model was an adequate fit. Model classification accuracy was also assured.

Linear regression was also carried out for some covariates associated with birth weight as continuous variables. These included maternal hemoglobin concentration (g/dl), maternal MUAC (cm) in the first or second trimester and delivery, BMI in the first trimester, and maternal DDS per 24 h. Multicollinearity, linearity, and interaction were checked among the variables considered in the model. The Scatter plot revealed linearity, and Durbin-Watson (<4) showed independence of the observations/data. The tolerance was greater than 0.1 and the variance of inflation was less than 10, indicating no multicollinearity.

Furthermore, fetal growth in the uterus strongly depends on maternal nutritional status and dietary practices, which could also be affected by the intervention. Considering this, a pathway and mediation analysis was conducted to assess the direct and indirect effects of the integrated nutrition intervention package on birth weight. In this case, the outcome variable is birth weight and the predictor for birth weight is the intervention. The nutritional status (MUAC) and dietary diversity score are mediators of the intervention impact. The pathway mediation was analyzed using PROCESS macro version 4.0 for SPSS designed by Hayes (30).

## Results

### Basic Socio-Demographic, Obstetric, and Lifestyle Characteristics of the Study Population

A total of 551 mother–baby pairs for intervention and 545 for controls were used in the analysis. Both groups were comparable in terms of the sex of the baby, maternal age, marital status, religion, education, occupation, family size, number of pregnancies, and birth spacing. However, the proportion of mothers taking alcohol, those who smoked, and those exposed to passive smoking during pregnancy were significantly higher in the control group than in the intervention group (Table 1).

**TABLE 1:** Socio-demographic, obstetric and lifestyle characteristics of the study population.

Variables	Intervention		Control		*P*-value*
	(*n* = 551)		(*n* = 545)		
			
	%	*n*	%	*n*	
**Sex of the baby**					
Male	43.9	242	47.0	256	0.31
Female	56.1	309	53.0	289	
**Age of the mother (years)**					
15–19	6.9	38	5.7	31	0.373
20–24	26.0	143	26.4	144	
25–29	28.5	157	26.4	144	
30–34	22.1	122	20.6	112	
35 and above	16.5	91	20.9	114	
**Marital status of the mother**					
Married/cohabitating	90.0	496	87.0	474	0.114
Single/divorced	10.0	55	13.0	71	
**Religion of the mother**					
Christian	94.7	522	96.0	523	0.559
Muslim	4.4	24	3.1	17	
Others	0.9	5	0.9	5	
**Level of education of the mother**					
None	11.4	63	12.1	66	0.297
Primary	63.0	347	62.4	340	
Secondary	24.5	135	22.9	125	
Tertiary	1.1	6	2.6	14	
**Occupation of the mother**					
Salaried employee	3.6	20	3.1	17	0.701
Self-employed	75.0	413	77.1	420	
Unemployed	21.4	118	19.8	108	
**Family size**					
2 to 4	58.4	322	56.3	307	0.091
5 to 7	34.7	191	39.3	214	
8 and above	6.9	38	4.4	24	
**Number of pregnancies**					
≤2	59.5	328	55.6	303	0.062
3 to 4	29.0	160	28.1	153	
>5	11.4	63	16.3	89	
**Birth spacing (months)*^a^***					
≤24	39.3	147	40.2	154	0.799
≥25	60.7	227	59.8	229	
**Taking alcohol or beer during pregnancy**					
No	87.8	484	82.8	451	**0.017**
Yes	12.2	67	17.2	94	
**Smoking during pregnancy**					
No	98.9	545	95.8	522	**0.001**
Yes	1.1	6	4.2	23	
**Passive smoking during pregnancy**					
No	92.4	509	84.2	459	**<0.001**
Yes	7.6	42	15.8	86	

^a^Total intervention = 374; total controls = 383, Remaining are first pregnancy. *Chi-square test was used to compare the proportions. Bold p value indicates significant association at p value ≤0.05.

### Maternal Nutritional and Birth Weight Status by Study Group

Table 2 shows the nutritional status of the mothers during pregnancy and the birth weight of their newborns. A significantly higher proportion of low birth weight (*p* < 0.001) was observed among the control group (10.3%) compared to the intervention group (3.4%). There was a significant variation in maternal anemia status, where anemia was more in the control group than intervention group (23.7% vs. 10.5%; *p* < 0.001). The proportion of maternal MUAC less than 23 cm at delivery was significantly higher (*p* < 0.001) among the control group (14.5%) compared to the intervention group (3.4%). Similarly, MUAC less than 23 cm during first/second trimester was significantly higher (*p* < 0.001) in the control group (18.2%) compared to the intervention group (4.5%). The average MUAC difference was significantly more among the intervention group compared to the control group (*p* = 0.020). BMI less than 18.5 kg/m^2^ in the first trimester was also significantly lower (*p* = 0.010) in the intervention (1.8%) than the control (5.5%) group. The average weight gain between the first and third trimester was significantly higher among the intervention group than that in the control group (*p* < 0.001). Although the average weight gain between the first and third trimesters was higher in the intervention group, there was no statistically significant difference (*p* = 0.119). Similarly, the proportion of maternal dietary diversity scores less than 5 out of 9 food groups was higher in the control group than the intervention group (25.7 vs. 18.3%; *p* = 0.003).

**TABLE 2:** Maternal nutritional and birth weight status by study group.

Variables	Intervention (*n* = 551)		Control (*n* = 545)		*P*- value
			
	%	*n*	%	*n*	
**Birth weight of the newborn baby**					
Normal birth weight (≥2,500 gram)	96.6	532	89.7	489	<0.001^φ^
Low birth weight (<2,500 gram)	3.4	19	10.3	56	
Birth weight (mean ± SD)	3216.34 ± 464.35		2997.34 ± 485.06		<0.001^ψ^
**Maternal anemia status**					
Normal (Hb ≥ 11 g/dl)	89.5	493	76.3	416	<0.001^φ^
Anemic (Hb < 11 g/dl)	10.5	58	23.7	129	
Maternal hemoglobin concentration (mean + SD)	12.65 ± 1.24		12.10 ± 1.48		<0.001^ψ^
**Maternal MUAC (cm) at delivery**					
≥23	96.6	532	85.5	466	<0.001^φ^
<23	3.4	19	14.5	79	
Maternal MUAC (mean + SD)	25.30 ± 1.77		24.18 ± 2.03		<0.001^ψ^
**Maternal MUAC (cm) in the first/second trimester*^a^***					
≥23	95.5	429	81.8	363	<0.001^φ^
<23	4.5	20	18.2	81	
Maternal MUAC (mean + SD)	25.46 (1.88)		24.30 (2.14)		<0.001^ψ^
**Maternal MUAC (cm) mean difference between first/second trimester and delivery**	0.84 ± 1.74		0.59 ± 1.42		0.020 ^ψ^
**Maternal BMI in first trimester*^b^***					
BMI < 18.5 kg/m^2^	1.8	7	5.5	25	0.010^φ^
BMI ≥ 18.5 kg/m^2^	76.1	296	76.4	349	
BMI > 25.0 kg/m^2^	22.1	86	18.2	83	
Maternal BMI in first trimester (mean + SD)	23.46 ± 2.97		22.73 ± 2.78		<0.001^ψ^
**Average weight gain**					
Average weight in first trimester [SD]*^c^*	61.86 (9.15)		58.58 (7.72)		<0.001^ψ^
Average weight in third trimester [SD]*^d^*	66.74 (9.52)		63.36 (7.75)		<0.001^ψ^
Average weight gain between third and first trimester [SD]*^e^*	4.91 (2.13)		4.78 (2.07)		0.394^ψ^
**Maternal dietary diversity score (DDS) (24 h)**					
High (≥5 DDS)	81.7	450	74.3	405	0.003^φ^
Low (<5 DDS)	18.3	101	25.7	140	
Maternal DDS (mean + SD)	5.791 (1.60)		5.49 (1.74)		0.003^ψ^

*^φ^Chi-square test was used to compare proportions. ^ψ^Means were compared using independent t test. ^a^Intervention = 449; Control = 444. ^b^Intervention = 389; Control = 457. ^c^Intervention = 382; Control = 452. ^d^Intervention = 389; Control = 460. ^e^Intervention = 389; Control = 452; The weight gain is an estimate between the weight taken any time within third trimester and first trimester using ANC records. BMI, body mass index; Hb, hemoglobin; Kg, Kilo-gram; MUAC, mid-upper arm circumference; SD, standard deviation.*

The mean birth weight was 3,216.34 g in the intervention group while it was 2,997.34 g in the control group. This difference was significant by 219 g more in the intervention group (*p* < 0.001). It was also observed that mean maternal hemoglobin concentration, maternal MUAC during the first/second trimester and during delivery, BMI in the first trimester, and dietary diversity scores, were significantly (*p* < 0.01) higher among the intervention group compared to the control group (Table 2).

### Bivariate and Multivariate Analysis: Socio-Demography, Obstetric, Lifestyle, and Other Characteristics Associated With Low Birth Weight

The following variables were found to be significant in bivariate analysis: level of education of the mothers (*p* = 0.001), passive smoking during pregnancy (*p* < 0.001), and maternal MUAC (*p* < 0.001). Although the proportion of LBW was lower (5.8%) among those aged 15–19 years compared to those 35 years and above (10.2%), there was no significant difference (*p* = 0.274). After considering/adjusting all variables with a *p*-value less than 0.2 together in multivariate analysis, passive smoking during pregnancy (AOR = 4.34; 95%CI = 2.64–7.15; *p* < 0.001) and maternal MUAC less than 23 cm (AOR = 2.95; 95%CI = 1.66–5.24; *p* < 0.001) were found to be independent risk factors for LBW (Table 3).

**TABLE 3:** Bivariate and multivariate analysis: Socio-demography, obstetric, lifestyle, and nutritional status associated with LBW.

Variables	Bivariate analysis				Multivariate analysis	
		
	LBW, %(n)	NBW, %(n)	COR (95%CI)	*P*- value	*^a^*AOR (95%CI)	*P*- value
**Sex of the baby**						
Male	8.8 (44)	91.2 (454)	1.14 (0.74–1.75)	0.56		
Female	7.9 (47)	92.1 (551)	1.00			
**Age of the mother (years)**						
15–19	5.8 (4)	94.2 (65)	0.54 (0.18–1.63)	0.274	0.57 (0.14–2.27)	0.427
20–24	11.1 (32)	88.9 (255)	1.1 (0.61–1.97)	0.749	1.36 (0.63–2.94)	0.432
25–29	7.0 (21)	93.0 (280)	0.66 (0.35–1.24)	0.193	0.84 (0.38–1.87)	0.673
30–34	13 (5.6)	94.4 (221)	0.52 (0.25–1.06)	0.071	0.57 (0.25–1.32)	0.189
35 and above	21 (10.2)	184 (89.8)	1.00		1.00	
**Marital status of the mother**						
Married/Cohabitating	7.9 (77)	92.1 (893)	0.69 (0.38–1.26)	0.227		
Single/divorced	11.1 (14)	88.9 (112)	1.00			
**Level of education of the mother**						
None	17.1 (22)	82.9 (107)	0.35 (0.18–0.68)	**0.002**	1.13 (0.52–2.46)	0.76
Primary	7.3 (50)	92.7 (637)	0.38 (0.22–0.66)	**<0.001**	2.31 (0.90–5.92)	0.081
Secondary and above	6.8 (19)	93.2 (261)	1.00		1	
**Occupation of the mother**						
Salaried employee	2.7 (1)	97.3 (36)	0.30 (0.04–2.33)	0.251		
Self-employed	8.5 (71)	91.5 (762)	1.02 (0.60–1.72)	0.956		
Unemployed	8.4 (19)	91.6 (207)	1.00			
**Family size**						
2 to 4	8.7 (55)	91.3 (574)	5.85 (0.79–42.98)	0.083	5.81 (0.73–46.01)	0.096
5 to 7	8.6 (35)	91.4 (370)	5.77 (0.78–43.00)	0.087	6.24 (0.79–48.93)	0.082
8 and above	1.6 (1)	98.4 (61)	1.00		1.00	
**Number of pregnancies**						
≤2	9 (57)	91	1.27 (0.65–2.49)	0.481		
3 to 4	7.3 (23)	92.7	1.02 (0.48–2.14)	0.965		
>5	7.2 (11)	92.8				
**Taking alcohol or beer during pregnancy**						
Yes	11.8 (19)	88.2 (142)	1.60 (0.94–2.74)	0.084	1.19 (0.59–2.40)	0.622
No	7.7 (72)	92.3 (863)	1.00		1.00	
**Smoking during pregnancy**						
Yes	17.2 (5)	82.8 (24)	2.38 (0.88–6.38)	0.086	0.68 (0.19–2.45)	0.555
No	8.1 (86)	91.9 (981)	1.00		1.00	
**Passive smoking during pregnancy**						
Yes	22.7 (29)	77.3 (99)	4.28 (2.63–6.97)	**<0.001**	4.37 (2.54–7.51)	<0.001
No	6.4 (62)	93.6 (906)	1.00		1.00	
**Maternal anemia status at delivery**						
Anemic (Hb < 11 g/dl)	9.6 (18)	90.4 (169)	1.22 (0.71–2.10)	0.472		
Normal (Hb ≥ 11 g/dl)	8.0 (73)	92.0 (836)	1.00			
**Maternal MUAC at delivery**						
<23 cm	19.4 (19)	80.6 (79)	3.09 (1.78–5.39)	**<0.001**	3.08 (1.67–5.66)	<0.001
≥23 cm	7.2 (72)	92.8 (926)	1.00		1.00	
**Maternal MUAC in the first/second trimester**						
<23 cm	16.8 (17)	83.2 (84)	4.19 (0.57–31.17)	0.161	1.23 (0.49–3.09)	0.659
≥23 cm	7.2 (57)	92.8 (735)	1.00		1.00	
**Maternal dietary diversity score (DDS) (24 h)**						
Low (<5 DDS)	10.8 (26)	89.2 (215)	1.47 (0.91–2.37)	0.115	0.95 (0.52–1.73)	0.865
High (≥5 DDS)	7.6 (65)	92.4 (790)	1.00		1.00	

*^a^AOR is adjusted against those variables with p value less than 0.200 during bivariate analysis. COR, crude odds ratio; AOR, adjusted odds ratio; CI, confidence interval. Bold p value indicates significant association at p value ≤0.05.*

### Effectiveness of Integrated Nutrition Intervention on Low Birth Weight

After controlling for the potential confounding against maternal nutritional status using multivariable logistic regression (backward conditional method), newborns in the intervention group were found to have a significantly lower risk of low birth weight (AOR = 0.23; 95%CI = 0.12–0.43; *p* < 0.001). Moreover, the multiple linear regression confirmed that the intervention group was significantly associated with higher birth weight (β = 0.16; 95%CI = 0.09–0.22; *p* < 0.001) (Table 4).

**TABLE 4:** Regression analysis for the effectiveness of integrated nutrition intervention on low birth weight.

Study group	Logistic regression				Linear regression			
		
	*^a^*AOR	95%CI		*P*- value	*^b^*Standardized beta coefficient	95%CI		*P*- value
						
		Lower	Upper			Lower	Upper	
Intervention group	0.23	0.12	0.43	**<0.001**	0.16	0.09	0.22	**<0.001**
Control group	1.00							

*^a^AOR (Adjusted odds ratio): adjusted for maternal age, level of education, family size, alcohol consumption, smoking, passive smoking, dietary diversity (<5 DDS), and MUAC < 23 cm. ^b^Standardized beta coefficient: adjusted for covariates of maternal hemoglobin concentration and maternal MUAC, BMI, and DDS per 24 h. MUAC: mid-upper arm circumference; CI, confidence interval. Bold p value indicates significant association at p value ≤0.05.*

### Pathway and Mediation Analysis for the Direct and Indirect Effects of the Intervention on Birth Weight

The path analysis also showed similar results with multiple linear regression. The average birth weight in the intervention group was significantly higher compared to the control group (β = 0.23; 95%CI = 0.18–0.28; *p* < 0.001). The direct effect of the intervention on birth weight was 0.17 (β = 0.17; 95%CI = 0.10–0.22; *p* < 0.001) while the indirect effect was 0.06 (β = 0.06; 95%CI = 0.04–0.10; *p* < 0.001). The main indirect mediator among the maternal nutritional indicators was MUAC (β = 0.05; 95%CI = 0.03–0.07; *p* < 0.001) (Table 5).

**TABLE 5:** Pathway and mediation analysis for the direct and indirect effects of the intervention on birth weight.

Variable	Standardized estimate	95% CI		*P*- value
			
		Lower	Upper	
Overall total effect of the intervention on birth weight	0.23	0.18	0.28	<0.001
Direct effect of intervention on birth weight	0.17	0.10	0.22	<0.001
Total indirect effect of the intervention on birth weight	0.06	0.04	0.10	<0.001
Indirect effect 1	0.05	0.03	0.07	<0.001
Indirect effect 2	0.005	–0.006	0.017	0.394
Indirect effect 3	0.006	0.001	0.013	0.025
Effect of hemoglobin concentration on birth weight	0.02	–0.03	0.08	0.386
Effect of MUAC on birth weight	0.23	0.17	0.28	0.003
Effect of maternal DDS on birth weight	0.07	0.001	0.12	0.023
Effect intervention on hemoglobin concentration	0.20	0.14	0.25	0.001
Effect of intervention on MUAC	0.24	0.18	0.29	0.001
Effect of intervention on maternal DDS	0.09	0.03	0.16	0.004

*Indirect effect 1, Intervention effect on birth weight through maternal MUAC. Indirect effect 2, Intervention effect on birth weight through maternal hemoglobin. Indirect effect 3, Intervention effect on birth weight through maternal DDS. MUAC, mid-upper arm circumference; DDS, dietary diversity score.*

## Discussion

The findings of this study revealed a significant improvement in birth weight among babies born to pregnant women who participated in this integrated maternal nutrition intervention program. The prevalence of low birth weight was decreased by 66.99% in the intervention group compared to the control group. Similarly, the mean birth weight of the newborn babies in the intervention group was 219 g higher compared to the control group. This result is similar to the recent studies done on nutrition education and counseling intervention in Ethiopia (31) and Kenya (32). However, it is challenging to compare it with other studies due to the combined interventions used in this study.

After adjusting for potential confounders, multiple logistic and linear regression demonstrated that the intervention was significantly associated with a reduction in low birth weight. This could be the result of the different combined interventions that contributed to an improved maternal nutritional status that in turn reduced low birth weight. However, some reviews and studies found mixed or varying results regarding the effectiveness of standalone interventions on maternal nutritional status and birth weight. For example, some reviews on maternal nutrition education and multiple micronutrient interventions showed a reduced risk of low birth weight (23, 25, 33, 34), while other reviews on only nutrition education intervention showed no or limited effects (3, 35–37). In addition, other single vitamin or mineral supplementation interventions, including vitamin A (38), folic acid supplementation (39, 40), iron (24), iodine (41), and zinc (42), marine oil, and fatty acid supplementation (43), were not found to be significantly associated with a reduction in LBW.

Generally, WASH intervention, as a combined intervention, is found to be more effective on infant nutritional status than the single WASH intervention (44, 45). Women’s economic empowerment (social safety nets) and cash transfer were associated with improved nutritional status and birth weight (37, 46). Though there is limited reporting of the impact of the agricultural intervention on birth weight (47), it is believed to produce a high effect when implemented within the other components (social safety nets and educational programs) (20).

In light of the above, this study confirms that there could be a synergic effect of the integrated nutrition intervention package. There was a significant direct effect of the intervention on birth weight (β = 0.16; *p* = 0.001). Maternal nutrition education and counseling significantly lead to increased and improved dietary practices among women (48, 49). The economic strengthening compounded with nutrition education may contribute to enabling women to use and readjust resources to take adequate and good quality diet, which in turn improves gestational weight gain and growth of the fetus (50). This was more effective when it is combined with agricultural intervention and nutrition education (20).

Overall, the combined intervention may enhance access and use of different health services, increase food security in the household, improve maternal nutrition knowledge, and safe food preparation, improve access to clean water and sanitation, empower women and enhance behavior change toward healthy practices. All of these may be among the possible reasons for the direct effect of the combined intervention toward improving birth weight. Therefore scaling up a range of multi-sectoral interventions during pregnancy could lead to increased birth weight (19, 20).

The study also found that the indirect effect of the intervention through maternal MUAC and maternal dietary intake practices significantly improved birth weight. Similarly, a systematic review on the effect of a nutrition intervention during pregnancy indicated an indirect effect of nutrition intervention on birth weight (3). A few other studies also reveal that dietary diversity among pregnant women can be improved through nutrition education, and this can in turn can reduce the risk of low birth weight (31, 51). Various studies have reported a strong relationship between maternal MUAC and birth weight, whereby women who delivered LBW babies were associated with low MUAC values (1, 28, 52).

### Strengths and Limitations

The strengths of this study are adequate sample size, well-organized post-intervention quasi-experimental design, and long duration impact of program evaluation (5 years). Moreover, to the best of our knowledge, this is the first study in Rwanda to report the effectiveness of integrated nutrition intervention on birth weight. However, the study had some limitations. First, we used only end-line post-program evaluation, which limits the ability to appreciate the trend of the nutritional indicators during and before pregnancy. Secondly, the lack of randomization to minimize some concealed confounding bias. Thirdly, it is difficult to know which component of the combined intervention contributes more to improving birth weight as the intervention was delivered as a package. Lastly, the 24-h food frequency before labor started was assessed after delivery, which could have been subject to some recall bias.

## Conclusion

The results of this study have shown that an integrated nutritional intervention package, including nutrition education/counseling, agriculture enhancement, economic strengthening through social safety nets, and access to WASH services, can significantly improve birth weight. Therefore, multiple sectors and stakeholders should intensify the different strategies mentioned above to address the diverse and complex determinants of maternal malnutrition to improve their nutritional status and in turn reduce the risk of low birth weight.

## Data Availability Statement

The raw data supporting the conclusions of this article will be made available by the authors, without undue reservation.

## Ethics Statement

The studies involving human participants were reviewed and approved by the Institutional Review Board (IRB) of the University of Rwanda College of Medicine and Health Sciences (CMHS). Written informed consent to participate in this study was provided by the participants’ legal guardian/next of kin.

## Author Contributions

MH designed and carried out the research and analyzed and wrote the manuscript. CM, AA, and MU participated in the design, discussion, and provided critical comments on the manuscript. MM provided critical comments on the manuscript. All authors have read and approved the manuscript for publication.

## Conflict of Interest

The authors declare that the research was conducted in the absence of any commercial or financial relationships that could be construed as a potential conflict of interest.

## Publisher’s Note

All claims expressed in this article are solely those of the authors and do not necessarily represent those of their affiliated organizations, or those of the publisher, the editors and the reviewers. Any product that may be evaluated in this article, or claim that may be made by its manufacturer, is not guaranteed or endorsed by the publisher.

## Abbreviations

AOR: adjusted odds ratio
BMI: body mass index
CI: confidence interval
DDS: dietary diversity score
Hb: hemoglobin
LBW: low birth weight
MUAC: mid-upper arm circumference
SPSS: Statistical Package for the Social Sciences
USAID: United States Agency for International Development
WASH: Water, Sanitation, and Hygiene.
