# Supplementary material for: Effectiveness of Integrated Maternal Nutrition Intervention Package on Birth Weight in Rwanda
Source: Front Nutr. 2022 Jul 22;9:874714. doi: 10.3389/fnut.2022.874714 (PMC9353189; doi:10.3389/fnut.2022.874714)
Supplement: Supplementary file 1 [file Data_Sheet_1.pdf]

Description of the integrated nutrition intervention package

Intervention Group	Control Group
**Nutrition-specific component**	
**Nutrition education and counselling:** In the intervention group the pregnant women received additional nutrition education and counselling by Community Health Workers (CHWs) and nutritionists. At the beginning, the nutritionists and CHWs in charge received an initial five-day training on the module of counselling guide from the program implementers. Then the CHWs in charge in turn trained CHWs at Village level. The trained nutritionists counselled the pregnant women during regular antenatal care visits that lasted about 30 to 45 minutes each. In addition, the CHWs gave education and counselling at the household level. They also received in-service training on a monthly basis. The nutritionists and CHWs in charge received regular refresher training every 6 months to one year and were supervised and evaluated on a monthly basis using a competency checklist. This evaluation criteria was based on the counseling guide provision, preparedness and accuracy of the whole content and ability to properly respond to questions.<br>The main contents of the educational and counseling guide were (1) to eat one extra small meal or "snack" (extra food between meals) each day to provide energy and nutrition for the mother and her growing baby; (2) to eat the best nutritious foods available, including milk, fresh fruit and vegetables, meat (including organ meat like liver, heart and kidney), fish, eggs, grains, groundnuts, peas and beans; (3) to drink plenty of liquids; (4) advice not to take tea or coffee with meals as they can interfere with the body's use of the foods; (5) to limit the amount of coffee taken during pregnancy (6) to take iron and folic acid tablets to prevent anemia during pregnancy; (7) to use iodized salt (8)) to attend antenatal care at least 4 times during pregnancy, beginning during the first 3 months; (9) to take de-worming tablets to help prevent anemia; (10) to prevent malaria by sleeping under an insecticide-treated mosquito net every night.	In the control group, pregnant women only received counselling about healthy eating as well as daily oral iron and folic acid supplementation. These are the standard nutrition care practices adopted from WHO ANC model (WHO Recommendations on Antenatal Care for a Positive Pregnancy Experience: Summary. Geneva, Switzerland: WHO; 2018. Licence: CC BY-NC-SA 3.0 IGO.) This care was delivered by nurses at the health facilities to those pregnant women attending ANC.
**Nutrition-sensitive components**	
1. **Promotion of increased agricultural productivity** This involved promotion of agricultural productivity through implementation of Bio Intensive Agriculture Techniques (BIATs), Farmer field learning school (FFLS), promotion of bio fortified crops and small livestock in all Villages from the five districts. The main activities included training on crop disease/pest control and improving the soil fertility, supplying and promoting indigenous vegetables and bio-fortified crops (Orange Fleshed Sweet Potato, Iron Rich beans, Quality Protein Maize and orange maize), promoting agriculture activities in urban areas using bags in case of no land, provision of small livestock	The control group did not receive this intervention

	(chicken, rabbit, goats and pigs) and sensitization on consumption of home garden produced through meetings and monitoring field visits.	
2.	**Promotion of financial literacy and economic resilience:** The *Gikuriro* program enhanced and promoted Saving and Internal Lending Communities (SILC) Groups approach as a way of responding to household financial problems that prevent them from attaining better nutrition outcomes. This was a holistic community-based, user-owned and self-managed savings method that offers a conducive environment for poor households to save and borrow to increase their income. Moreover, the main purpose was to teach them the basic financial management skills to better manage their existing resources. SILC was a savings group approach developed by Catholic Relief Services (CRS) that promoted accessible, transparent and flexible social protection mechanisms. The main activities of SILC under *Gikuriro* were as follows:<br>First, training of trainers was conducted to *Gikuriro* sub-partners (the in charge of economic strengthening and project coordinators) and district staff cooperatives. Then the trained staff trains the sector cooperative officers where they would identify community volunteer called field agents. They are identified within the community using a checklist, after which they are interviewed by CRS. They are trained to form and manage SILC groups which will ensure that all beneficiaries are embraced in the SILC groups and receive high quality financial services. Then the field agents sensitized the people about SILC and form groups. Members select each other based on characteristics of trustworthiness, honesty, reliability, and punctuality. One field agent supervises 10 groups of which one group consists of 25 to 30 members. Then after a group is formed, they decide how often to meet, the minimum contribution amount and how long to function and discuss about internal rules and select management committee. The group also selects Chairperson, Secretary, Treasurer and a Money Counter from its members. The frequency of meetings and contributions is on weekly basis. The maximum contribution does not exceed five times the minimum contribution. The cycle of savings and lending is 12 months.<br>Pooled contributions create a loan fund for members to be repaid with interest and a social fund to help members with emergency situations. By the end of the cycle, all loans are repaid. Accumulated savings and interest earnings are paid out in proportion to members’ contributions relative to the amount that has been invested by each member throughout the cycle. After pay-out, the group may disband or decide to continue for another cycle and may invite new members to join. During the year, more focus is put on monitoring of established SILC groups to ensure savings are bringing a change in the nutritional outcomes at target HHs. This enables the poor to build up useful lump sums without incurring excessive debt or interest charges. The goal is	No such intervention was given to the control group

	to help these households better manage their existing resources by teaching them basic financial management skills.	
3.	**Water, Sanitation and Hygiene (WASH) services:** This intervention was aimed at improving WASH behavior in the community to avoid nutrition problems resulting from poor WASH behaviors using Community Based Environmental Health Promotion Program (CBEHPP) approach through Community Health Clubs (CHC) at Village level. CBEHPP is a hygiene behavior change approach to reach communities and empower them to identify their personal and domestic hygiene and environmental health-related problems (including access to safe drinking water for instance having boreholes, water kiosks, extending water pipelines to each Village and improved sanitation) and to solve them. CHC and a demonstration site at every Village is formed and initiated. They conduct 20 weekly community health club sessions using high-quality instructional materials. The CHCs are responsible of ensuring that levels of hygiene are monitored, together with the CHW facilitator, who visit each household to observe the household sanitation and environmental conditions. These observations, known as a 'household inventory' are conducted on a regular basis. Besides a Chairperson and Secretary are elected, who keep a register of attendance of the members.	The control group did not receive such intervention
